# Chemical Formulation and Characterization of Complementary Foods from Blend of Orange-Fleshed Sweet Potato, Brown Teff, and Dark Red Kidney Beans

**DOI:** 10.1155/2020/4803839

**Published:** 2020-05-14

**Authors:** Tesfay Araro, Feyera Gemechu, Aselefech Wotango, Tarekegn Esho

**Affiliations:** ^1^Department of Industrial Chemistry, Addis Ababa Science and Technology University, Addis Ababa, Ethiopia; ^2^Department of Food Engineering, Addis Ababa Science and Technology University, Addis Ababa, Ethiopia

## Abstract

Consumption of nutritionally inadequate diets results in infant malnutrition. This study is aimed at formulating complementary foods from blend of orange-fleshed sweet potato, brown teff, and dark red kidney beans for infants aged 6–23 months. The Design-Expert 6.0.8 Software was used to formulate flour blends. Proximate, mineral, and antinutrient characterizations of flour blends were determined by using standard methods. The crude protein, crude fat, carbohydrate, and energy contents of raw flour blends were varied from 4.90–14.25%, 1.63–1.99%, 67.10–76.29%, and 339.07–343.63 Kcal/100 g, respectively. The crude protein, crude fat, carbohydrate, and energy contents of extruded blends were varied from 3.65–12.41%, 0.16–0.31%, 72.66–83.96%, and 343.07–356.74 Kcal/100 g, respectively. The crude protein, crude fat, carbohydrate, and energy contents of drum-dried blends were varied from 4.45–14.08%, 1.21–1.70%, 69.30–80.45%, and 347.20–356.57 Kcal/100 g, respectively. The products meet the recommended daily intake of protein (5.2–10.9 g), carbohydrate (≥65 g), energy (202–894 Kcal), and potassium (60–160 mg) for infants. However, the products are in short of meeting some of the recommendations given by international standards regarding daily intake of fats and minerals. Therefore, it requires some enhancement by including studied amount of butter and other micronutrient dense foodstuffs.

## 1. Introduction

Consumption of nutritionally poor complementary foods and inappropriate feeding practices are the main contributing factors to the development of childhood malnutrition in many developing countries including Ethiopia [1]. Traditional infant foods made of cereals are low in protein, fat, vitamin A, zinc, iron, and high in antinutritional factors that reduce nutrients and mineral bioavailability [2]. Good quality complementary foods must be rich in macro/micronutrients and low in antinutrient content and viscosity [3, 4]. In Ethiopia, every year, one million children under the age of five years die because of protein-energy malnutrition [5].

Malnutrition can also result from diseases that interfere with the body's ability to use the nutrients consumed [6]. The body of malnourished children is weak to resist infections [7]. Stunting reveals chronic undernutrition during the most critical periods of growth and development in early life. Globally, undernutrition is responsible for at least 35% of deaths in children aged under five [6]. Worldwide, 165 million children below five years of age are affected by undernutrition, of which 26% are stunted [8]. The alleviation trial to reduce the prevalence of malnutrition in developing countries has been slow [9]. Ethiopia's achievement is also limited with an annual reduction of 1.3% [10].

Breast milk is rich in nutrients but not enough to meet the macro and micronutrient requirements of infants after six months of age. After six months of age, nutritional composition of breast milk becomes inadequate to meet the infant's nutritional requirement [11]. Hence, complementary foods need to be formulated and introduced in a child's diet after six months while continuing breastfeeding up to twenty-four months and beyond. Complementary foods should be of appropriate nutritional quality and energy content to complement the nutritionally required chemicals that are obtained from breast milk for infants and younger children [12].

Nutritionally, an adequate diet is very important for infants' growth and development. The period from birth to twenty-three months is especially important for optimal physical, mental, and cognitive growth, health and development. Unluckily, this rage of age is often known by protein-energy and micronutrient deficiencies that interfere with optimal growth [13]. Most foods that developed in different countries to provide nutrient-dense complementary foods to meet the nutritional needs of infants and young children are based on local foodstuffs and blended with legumes to provide protein [14]. In this study, orange-fleshed sweet potato, brown teff, and dark red kidney beans were used to formulate complementary food from their flour blend. Sweet potato as a complementary food has been identified as a sustainable product for supplementing the nutritional needs of babies in developing countries [15]. Orange-fleshed sweet potato is high in carbohydrate and vitamin A content but low in crude protein and fat contents [16]. Hence, it needs to be complemented with legumes and cereals when being used in complementary foods. Legumes such as red kidney beans are an important source of dietary proteins. They play a significant role in human nutrition by complementing other foods such as wheat and other cereals [17]. From cereals, brown teff is rich in iron content and can combat iron deficiency (anemia) [18]. Therefore, a blend of orange-fleshed sweet potato (OFSP), brown teff (BT), and dark red kidney beans (DRKB) in the formulation of a complementary food can increase the nutritional composition of the food.

## 2. Materials and Methods

### 2.1. Sample Collection and Preparation

Four kilograms of dark red kidney beans ((*Phaseolus vulgaris*) (dark red kidney (*DRK*) variety)) were collected from the Melkassa Agricultural Research Center, 3 Kg of brown teff ((*Eragrostis Teff*) (*Dz-01-99* or *Asgori* variety)) were collected from the Debre-zeit Agricultural Research Center, 12 Kg of orange-fleshed sweet potatoes ((*Ipomea batatas* (*L.*) *Lam.*) *Kulfo* variety)) were collected from the Hawassa Agricultural Research Center, and commercial complementary food (*faffa*) was purchased from the supermarket of Addis Ababa city as a control.

Brown teff samples were sorted for extraneous materials and then washed with tap water until all soil and other unwanted particles were removed completely. This was followed by drying the teff grains by using sunlight for 8 h at 25°C. The drying was done by spreading on aluminum foils and putting on the table and kept at an elevated position to keep away from dust contamination [19]. Similarly, kidney beans were washed with tap water and dried by using sunlight for 8 h at 25°C. Orange-fleshed sweet potatoes were washed manually with tap water, peeled with kitchen peeling machine, and sliced into 1 cm thick slices by using a vegetable slicer and washed with water. Finally, the sliced samples were dried in a Memmert universal oven (Model-UF 260, Memmert GmbH+CO.KG, Germany) at 60°C for 12 h [20].

The dried brown teff, orange-fleshed sweet potato, and dark red kidney bean samples were milled by using a laboratory scale milling machine (0.5 mm standard sieve). Weight of brown teff, dark red kidney beans, and orange-fleshed sweet potato flour were 2.85 Kg (95%), 3.68 Kg (92%), and 4.20 Kg (35%), respectively. All samples were packed in airtight amber bottles, labeled, and stored at room temperature until the next analysis and blend formulations were carried out. All these activities were done in the Food Process Engineering Department's Cereal Laboratory, Addis Ababa Science and Technology University.

### 2.2. Chemical Formulation of Complementary Flour Blends

To formulate different flour blends, the Design-Expert 6.0.8 Software (D-Optimal mixture design) was used. The formulated blends were expected to provide the recommended daily intake of the macro and micronutrients [21–23]. Seven representatives were selected from the formulations. The formulated complementary flour blends were packaged in airtight amber bottles, labeled, and stored at room temperature.

### 2.3. Extrusion Process of Raw Complementary Flour Blends

The flour blends were extruded by using twin screw extruder (Model 838117.010, Brabender® GmbH and Co. KG and YOM 2016, Germany) in the following working conditions. The moisture content of the samples were 21%, and the temperatures were set as 60°C (for zone-6), 60°C (for zone-5), 80°C (for zone-4), 100°C (for zone-3), 120°C (for zone-2), and 145°C (for zone-1) from the feeder end to the die [24]. The diameter of the die was 3 mm, speed of screws was set at 150 rpm, the rate of feeding was set at 100 g/min, and the pressure was set at 2.4 bars. The extruded products were dried in an oven at 100°C for 20 min to remove extra moisture. After cooling at room temperature, the products were milled into fine flour and packed in airtight amber bottles at room temperature prior to the next analysis [24].

### 2.4. Drum-Drying Process of Raw Complementary Flour Blends

The formulated raw flour blend samples were drum dried by using a laboratory scale double drum drier (Model-FT 32-E, Armfield limited, France). 95% of water per weight of flour blend was added to each of the samples and kneaded into dough. The dough was poured slowly onto the drums. The pressure of steam was set at 2.5 bars. The temperature was set at 127°C, and the revolution of drums was set at 20 revolutions per minute (rpm) [25]. Thin dry films were produced after 15 min. The dried films were scraped off from the drums by means of a steel knife, collected, and milled into fine flour. Each complementary flour blends were packed for the next analysis and gruel preparation.

### 2.5. Determination of Proximate Chemical Composition of Each Ingredients and Formulated Flour Blends

Standard procedures of AOAC were used to determine the moisture, ash, crude protein, crude fat, and crude fiber content [26]. Available carbohydrate content was calculated by subtracting the summation of the percentage of moisture, ash, crude protein, crude fat, and crude fiber content from 100% [27]. Energy contents of all the formulated complementary flour blend samples were determined by calculation using Atwater's conversion factors [28, 29].

### 2.6. Mineral Analysis of Formulated Complementary Flour Blends

Mineral contents of the complementary raw, extruded, and drum-dried flour blends were determined by using the atomic absorption spectrometer (ZEEnit 700p, Analytik Jena, India) [30]. The methods were briefly described as follows: For wet digestion of the sample, exactly 1 g of the powdered sample was taken in digesting glass tube. Twelve milliliters of conc. HNO_3_ (69.0-72.0%, Central Drug House Pvt. Ltd.) was added to the flour blend samples, and the mixture was kept overnight at room temperature. Then 4.0 ml conc. perchloric acid (HClO_4_) (70%, Sisco Research Laboratories Pvt. Ltd.) was added to this mixture, and the process was continued. The temperature was started from 50 °C and increased up to 300 °C. The digestion process was completed in about 85 min as was indicated by the appearance of white fumes. The digest was left for 30 min to cool down. After cooling, the contents of the digestion tubes were poured to 100 ml volumetric flasks and the volumes of the contents were made to 100 ml with distilled water. The wet digested solutions were poured into plastic bottles and labeled correctly. The labeled digests were saved in a refrigerator at 4°C for mineral determination.

#### 2.6.1. Determination of Iron (Fe), Zinc (Zn), Calcium (Ca), Potassium (K), and Magnesium (Mg) Concentration

The digested samples kept in the refrigerator at 4°C were analyzed for mineral contents by Atomic Absorption Spectrophotometer [31, 32]. A corresponding hollow cathode lamp was used for each element. Before the determination of the concentration of minerals in each sample, the equipment was run for standard solutions ((iron (Fe), zinc (Zn), calcium (Ca), potassium (K), and magnesium (Mg) AAS standard solutions 1000 mg/l in nitric acid (Research-LAB Fine Chem Industries)) of each mineral. This activity is also used to check that the equipment is working properly. The dilution factor for all minerals except magnesium was 100. To determine magnesium concentration, further dilution of the original solution was done by using a 0.5 ml original solution, and enough distilled water was added to it to make the volume 100 ml. Also, to determine calcium concentration, 1.0 ml lithium oxide (Li_2_O) solution was added to the original solution to unmask calcium from magnesium. Calibration curves of standards were prepared. Concentrations of each mineral in test solutions were calculated from the calibration curve prepared by using the standard solutions of the respective mineral substances [33]. The concentrations were calculated using the formula:

(1) $$\begin{array}{c} \text{Mineral content }\left(\frac{\text{mg}}{100}\text{g}\right)=\frac{C\times D}{1000}\text{x }100, \end{array}$$

where *C* = concentration of the mineral in 1 g of sample (mg/Kg), 1000 = conversion factor (Kg into g), and *D* = Dilution factor.

### 2.7. Antinutritional Analysis of Formulated Complementary Flour Blends

#### 2.7.1. Phytate Determination

Centrifuge test tubes were washed and dried in an oven at 80°C. Stock solution of HCl (35.38%, Central Drug House Pvt. Ltd.) was used to prepare 0.2 N of HCl by using deionized water as a solvent. Accurately weighed 0.1 g of raw, extruded, and drum-dried flour blend samples was placed into test tubes, and 10 ml of 0.2 N HCl solutions was added to each sample. Then phytic acid was extracted from 0.1 g of raw, drums-dried, and extruded flour blend samples by using a mechanical shaker for 1 h at ambient temperature and centrifuged at 3,000 rpm for 30 min. Three milliliters of clear supernatant was used for phytate determination. Two milliliters of Wade reagent ((contained equal volume of 0.03% of FeCl_3_.6H_2_O (99.5%, Titan Biotech Pvt. Ltd.) and 0.3% of sulfosalicylic acid (≥99%, Sigma-Aldrich) in deionized water)) were added to 3 ml of clear supernatant, and the mixture was mixed using Vortex (Model VM-300p) for 5 sec. The absorbance of the sample solutions was measured at 500 nm using a UV-vis spectrophotometer (PerkinElmer, Model-Lambda 950, Indonesia). A series of standard solutions (5, 9, 18, 27, and 36 ppm) were prepared from 90 ppm of stock phytic acid ((analytical grade phytic acid sodium salt (≥90%, Sigma-Aldrich)) by using 0.2 N HCl solution as a solvent. Three milliliters of 0.2 N HCl and 2 ml of Wade reagent were added into a centrifuge test tube, which was used as a blank. Two milliliters of the Wade reagent was added to each test tube that contained 3 ml of standard solution. The solutions were mixed on a Vortex mixer for 5 sec. The mixtures were centrifuged at 3,000 rpm for 10 min, and the absorbance of the blank and standards was measured at 500 nm by using UV-visible spectrophotometer. The phytate concentration was calculated by subtracting the absorbance of the analyzed sample from the absorbance of the blank (3 ml of 0.2 N HCl + 2 ml of wade reagent) (Equation (2)). The amount of phytic acid was calculated by using a phytic acid standard curve, and the result was expressed as phytic acid in *μ*g/g. The calibration curve (absorbance versus concentration) was plotted, and the slope and intercept were found out [33–35].

(2) $$\begin{array}{c} \text{Phytic acid in }\frac{\mu g}{g}=\frac{\left[\left(A_{b}-A_{s}\right)\right]\left.-\text{Intercept}\right]}{\text{Slope X W X }3}\times 10, \end{array}$$

where *A*_*s*_ = Sample absorbance, *A*_*b*_ = Blank absorbance, *W* = Weight of sample, and 3 = Volume of Supernatant.

#### 2.7.2. Condensed Tannin Determination

One gram of raw, extruded, and drum-dried flour blend samples was weighed in centrifuge test tubes, and 10 ml of 1% HCl in methanol (99.9%, Biochemical synthesis services) was added into each test tube. Condensed tannins were extracted from flour blend samples by shaking on a mechanical shaker for 24 h at room temperature. Then, the solution was centrifuged at 1,000 rpm for 5 min. One milliliter of the supernatant was taken and mixed with 5 ml of vanillin-HCl reagent ((prepared by mixing equal volume of 8% concentrated HCl in methanol and 4% Vanillin (≥97%, Sigma-Aldrich) in methanol)) in another test tube. D-catechin (≥98%, Sigma-Aldrich) was used as a standard for condensed tannin determination. Sixty milligrams of D-catechin was weighed and dissolved in 100 ml of 1% HCl in methanol, which was used as a stock solution. A 0, 0.2, 0.4, 0.6, 0.8, and 1 ml of the stock solution were taken in different test tubes, and the volume of each test tube was adjusted to 1 ml with 1% HCl in 99.9% methanol. Zero milliliter of stock solution with 1 ml of 1% HCl in 99.9% methanol was used as a blank solution. Five milliliters of vanillin-HCl reagent was added into each test tube including a blank solution test tube. The solutions were kept for 20 min to complete the reaction. Then, the absorbances of the sample, blank, and standard solutions were measured at 500 nm by using UV-vis spectrophotometer. The calibration curve (absorbance versus concentration) was plotted, and the slope and intercept were found out [33, 34, 36, 37]. The concentration of condensed tannin was calculated by using the formula as indicated in Equation (3).

(3) $$\begin{array}{c} \text{Condensed Tannin in }\frac{mg}{g}=\frac{\left[\left(A_{s}-A_{b}\right)\right]\left.-\text{Intercept}\right]}{\text{Slope X d X w}}\times 10, \end{array}$$

where *A*_*s*_ = Sample absorbance, *A*_*b*_ = Blank absorbance, *w* = Weight of sample in gram, and *d* = Density of tannin stock solution (0.791 g/ml).

#### 2.7.3. Oxalate Determination

The concentration of oxalate was determined by AOAC, 2005 method [38]. The detail of the method is explained as follows. One gram of the samples was weighed into a conical flask (500 ml). Seventy-five milliliters of 3 M H_2_SO_4_ (98%, Loba Chemie Pvt. Ltd.) was added and stirred with a magnetic stirrer for 1 h and then filtered by using Whatman No. 1 filter paper. The filtrate or extract was collected with another conical flask. Twenty-five milliliters of the filtrate was measured and poured into a beaker (400 ml) and then titrated against 0.1 M KMnO_4_ (min. 99%, Alpha Chemika) solution that was preheated for 1 h in the water bath at 90°C. The titration was continued until the pink color appeared and persisted for at least 30 sec. The volume consumed was recorded. The oxalate content was given by the relationship that 1 ml of 0.1 M KMnO_4_ solution = 0.006303 g of oxalate [11, 33, 39]. The following formula (Equation (4)) was used to calculate the percentage of oxalate content in 100 g of flour sample.

(4) $$\begin{array}{c} \%\text{ Oxalate}=\frac{\text{titre}}{W}\times 0.006303\text{ x }100, \end{array}$$

where *W* = Weight of sample used and titre = volume of titrant (KMnO_4_) consumed.

### 2.8. Gruel Preparation and Viscosity Determination

Drum-dried samples were used to prepare gruels. A well-known maize-based complementary food (*faffa*) was used as the control sample in sensory evaluation. Two hundred fifty milliliters of water was added in a beaker (500 ml) and boiled at 85 °C on stove. Fifty grams of *faffa* flour was mixed with 50 ml of water in another beaker to form slurry. The slurry was poured into the boiling water. The resulting slurry was constantly stirred by using a wooden ladle until a paste was formed. The cooking process of food was completed around 15 min, and 20 g of sugar was added. The drum-dried flour blend gruel samples were prepared in the same manner. These gruel samples were ready to measure their viscosity and served to panelists for sensory assessment [40].

The viscosity of gruels was measured by using Brookfield viscometer (Model DVI+, Italy). Cooked gruels were placed in a water bath maintained at 40°C. The temperature 40°C is a temperature at which viscosity measurement was taken and serving temperature [41]. Brookfield viscometer was used to measure the gruels viscosity (in centipoises, cP) using spindle number 4 at a shear rate of 50 rpm [1].

### 2.9. Sensory Evaluation

**Figure 1:**
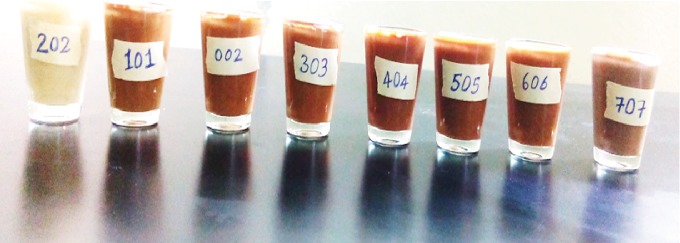
Images of gruel samples.

The formulated samples were made into gruels, using 1 : 6 ratios of flour and water [40]. The formulated blends were compared along with a commercial complementary food (*faffa*). In this study, a panel of 42 students (23 students from food science and 19 students from food process engineering) and 10 teaching staffs were involved. These students and teaching staffs have experience in sensory evaluation activities. The sensory evaluation was conducted in the Sensory Laboratory Room in the Food Science Department of the Addis Ababa Science and Technology University, where each of the panelists was requested to sit in separate positions to avoid interference. The samples were rated for the attributes such as color, smell, taste, mouthfeel/texture, after taste, and overall acceptability using seven points hedonic scale (1 = dislike very much through 4 = neither like nor dislike and 7 = like very much) [11]. These food samples were coded with 3 digits and served by using glass cups (40 ml) and disposable spoons in a random order for panelists to assess as shown in Figure 1. Water was given to panelists to rinse their mouth to avoid taste interference.

### 2.10. Statistical Analysis

All measurements were carried out in triplicate for each of the samples except mineral analysis and sensory evaluation. The mean and standard deviation (SD) were calculated. The data were analyzed by one-way analysis of variance (ANOVA) using SPSS version 24.0 software for windows. Values were presented as means of triplicates ± SD and compared using Duncan's multiple range tests [42]. A level of *P* < 0.05 was used to indicate significant differences among the samples.

## 3. Results and Discussion

### 3.1. Proximate Composition of Raw Materials

Proximate compositions of orange-fleshed sweet potato (OFSP), brown teff (BT), and dark red kidney beans (DRKB) are depicted in Table 1.

As explained in Table 1, the significant change was observed in the moisture contents of OFSP, BT, and DRKB flour. This change may be originated from sample size and mode of sample preparation methods. Researchers observed that higher moisture content could lead to food spoilage through increasing microbial action. The low moisture content of food remains an advantage in the storage and preservation of the nutrients [43]. The ash content of DRKB, OFSP, and BT flour were found to be 3.93%, 2.91%, and 2.32%, respectively. The ash content in a given food sample indicates the level of minerals present [44]. According to the other macronutrient compositions, DRKB was the major source of protein, OFSP was the major source of carbohydrate, and BT was a little bit better source of fat as shown in Table 1.

The OFSP protein (2.25%) of the current study is found to be higher in some cases (1%) [45] and lower in others (5.17%) [46]. Elsewhere report showed that the protein content of OFSP could be as high as 9.21 to 9.80% depending on its variety [47]. Some literature [45] reported a higher value of carbohydrate content (90.6%) than the current study, whereas others reported within a similar range (80–83.29%) [46, 47]. The difference may be originated from the variety and mode of cultivation [48]. The fat content obtained in this study (0.60%) is comparable with the reported one (0.70%), but the ash value (2.91%) is lower than the reported value (4.00%) [49]. The protein content of DRKB (18.01%) obtained in this study is lower than the reported protein value (21.99%) [50].

On the other hand, the fat content of DRKB (1.76%) is similar with previously reported value (1.50%) [51]. Protein (9.49%), ash (2.32%), carbohydrate (70.87%), and fat (2.77%) contents of BT were comparable with the reported contents of 10.5%, 3.10%, 73.10% [18], and 2.70%, respectively [52]. The difference observed in these chemical compositions may be due to differences in climate, mode of cultivation, and soil conditions under which they were cultivated [48].

### 3.2. Proximate Composition of Formulated Complementary Flour Blends

Proximate compositions of formulated complementary flour blends are depicted in Table 2.

Proximate contents in most of the raw, extruded, and drum-dried blends with the same blending ratios were significantly different (*P* < 0.05) as shown in Table 2. High protein content was observed in blends which had a high percentage of DRKB and BT flour in their flour composition as expected.

As shown in Table 2, moisture content of all extruded and drums dried complementary flour blends was significantly less than the moisture content of raw formulated complementary flour blends. It may be due to the high temperature of extrusion cooking and drum-drying processes. Such low moisture content of flours inhibits microbial activity and prolongs the shelf life of the flours [53]. Extruded complementary flour blends had significantly lower moisture content than drum-dried flour blends. This may be due to the higher temperature of the extrusion cooking process (varied from 60–145°C) than the drum-drying process (127°C).

Ash content of all extruded and drum-dried complementary flour blends (varied from 3.39–4.05%) meets the recommended amount in complementary foods (<5 g/100 g) [54]. Ash content of the currently developed products is higher than the ash content (varied from 2.11–3.02%) of composite flour blends of wheat, anchote, and soybeans [55]. The protein contents in extruded and drum-dried products varied from 3.65–14.08%. Both extruded and drum-dried products had significantly lower protein content (4.90–14.25%) than the raw products. Extruded complementary flour blends (3.65–12.41%) had significantly lower crude protein content than drum-dried complementary flour blends (4.45–14.08%). This effect of extrusion and drum-drying processes or reduction of protein content was may be due to the Maillard reaction, which can occur between alkaline amino acids and reducing sugars [56].

Assuming adequate breast milk intake, the amount of protein needed from complementary foods is 5.2 g/day at 6–8 months, 6.7 g/day at 9–11 months, and 9.1 g/day at 12–23 months of infants. Assuming inadequate breast milk intake, protein requirement is 9.1 g/day at 6–8 months, 9.6 g/day at 9–11 months, and 10.9 g/day at 12–23 months of infants [21, 57]. The protein content of DDb1 (5.97 g) meets the recommended intake of protein (5.2 g/day) for 6–8 months of infants and (6.7 g/day) for 9–11 months of infants with adequate breastfed. DDb4 (12.89 g) and DDb5 (14.08 g) have greater protein content than that of the daily recommended intake of protein (9.1 g/day) for 12–23 months of infants with adequate breastfed and for 6–8 months (9.1 g/day), 9–11 months (9.6 g/day), and 12–23 months (10.9 g/day) of infants with inadequate breastfed. In the current study, the protein content of DDb5 (14.08 g) meets the recommended amount (14.00 g/100 g) by Ethiopian Standards Agency (ESA) [58] but does not meet the recommended amount (15 g/100 g) by Codex Alimentarius Commissions (CAC) from complementary foods [59]. In terms of commercial infant foods, protein contents of current products are more appropriate than protein contents of *faffa* (17 g/100 g) and cerifam (18 g/100 g) for infants aged 6–23 months [60].

The protein content in DDb5 (14.08%) may be due to the higher proportion of DRKB (48%) and BT (39%) in its blended flour. This protein result is within the range of infant food (famix) (≥14%) [60] but higher than the reported protein content of local complementary foods in the Oromia Region (10.7%) and SNNP Region (11.2%) in Addis Ababa, Ethiopia [61]. In the current study, the protein content of DDb4 (12.89%) is found to be higher than the reported protein contents (varied from 7.68–8.56%) of maize-soybean blends [62]. Protein content of DDb5 (14.08%) is similar with the reported value of amaranth boiled plus sorghum boiled blend (14.00%) and extruded flour blend of red teff, OFSP, and soybeans (varied from 13.17–13.53%) in Ethiopia [19]. It is higher than the reported protein content (12.9%) of amaranth roasted, boiled plus sorghum boiled blend in Kenya, and protein contents (varied from 8.77–9.30%) of maize, OFSP, and haricot beans flour blends in Southern Ethiopia [21, 63]. Protein and carbohydrate content of DDb4 and DDb5 are higher than the reported protein (12.39% and 12.48%) and available carbohydrate content (56.07% and 58.92%) for extruded and drum-dried sweet potato-based complementary foods, respectively, and lower than the reported protein content (16.96%) of drum-dried OFSP, millet, and soybean flour blend complementary food in Ghana [20, 25].

A high fat content in a complementary food provides more energy to the infant. However, if it exceeds the desirable level, it would be disadvantageous for the stability of the product as the unsaturated fatty acids are vulnerable to oxidative rancidity that would shorten its shelf life [64]. On the other hand, a lower fat content in complementary foods results in poor energy density, which is of a big concern in complementary feeding [65]. The amount of fat in this study was varied from 0.16–0.31 g/100 g and 1.21–1.70 g/100 g in all extruded and drum-dried complementary flour blends, respectively (Table 2). Assuming adequate breast milk intake, the amount of fat needed from complementary foods is zero at 6–8 months, approximately 3 g/day at 9–11 months, and 9–13 g/day at 12–23 months [66]; and for inadequate breastfed infants is 20.5 g/day at 6–8 months, 22.9 g/day at 9–11 months, and 29.8 g/day at 12–23 months [21, 57]. In this study, fat contents are found to be higher than the recommended daily intake for infants aged 6–8 months and below for infants aged 9–11 months and 12–23 months with adequate breastfed. From drum-dried products, DDb4 (1.55 g/100 g) can fulfill at 3–4 servings per day the recommended daily intake of fat (3 g/day) for infants aged 9–11 months with adequate breastfed. All the extruded and drum-dried complementary flour blends contain below recommended daily intake of fat for infants with adequate breast fed aged at 12–23 months and inadequate breastfed aged at 6–23 months. The fat content of drums dried products is similar with the reported fat content of composite flour blends of wheat, anchote, and soybeans (varied from 1.50–1.76%) [55].

The crude fiber content of the supplementary food should not exceed 5 g/100 g dry matter [59, 67]. In the current study, the amount of crude fiber in all extruded and drum-dried complementary flour is found in optimum level (Table 2). During the extrusion cooking and drum-drying process, the fiber content was found to be lower than raw products (Table 2). This may be due to the applied high temperature and shearing force.

The recommended available carbohydrate content of complementary food products are ≥65 g/100 g [22]. Therefore, available carbohydrate content of all extruded and drum-dried complementary flour blends is in optimum range (Table 2). In developing countries, the expected energy intake for infants with adequate breastfed is approximately 202 Kcal/day at 6–8 months, 307 Kcal/day at 9–11 months, and 548 Kcal/day at 12–23 months [66]; and for infants with inadequate breastfed is 616 Kcal/day at 6–8 months, 686 Kcal/day at 9–11 months, and 894 Kcal/day at 12–23 months [21–23]. All extruded and drum-dried complementary flour blends are found to be above the lower limit of recommended daily intake of energy for infants aged 6–8 months (202 Kcal) and 9–11 months (307 Kcal) but below for infants aged 12–23 months (548 Kcal) with adequate breastfed. In addition to this, all the extruded and drum-dried complementary flour blends have lower energy content than the recommended daily intake of energy for infants with inadequate breastfed. This may be due to the corresponding lower values of crude fat in blends. However, all the products can meet the recommended daily intake of energy for infants aged 12–23 months with adequate breastfed at 2–3 servings per day, and it can also meet the requirement at this rate for infants 6–8, 9–11, and 12–23 months with inadequate breastfed. Energy levels which resulted in all current products are found to be higher than the reported composite flour blends of wheat, anchote, and soybeans (varied from 237.34–269.50 Kcal/100 g) [55].

### 3.3. Mineral Composition of Raw Complementary Flour Blends

Mineral compositions of raw complementary flour blends are depicted in Table 3.

As shown in Table 3, the concentration of calcium (Ca) (30.56 mg/100 g) in Rb1 was found to be the highest value than the rest raw complementary flour blends (they varied from 16.14–24.77 mg/100 g). The concentration of iron (Fe) (2.00 mg/100 g) was found to be the highest result than the other raw complementary flour blends. However, the Fe contents of all raw complementary flour blends except Rb1 were similar to each other (varied from 0.95–1.32 mg/100 g). The concentration of potassium (K) in Rb1, Rb2, Rb3, and Rb5 was found to be higher than the others (Rb4, Rb6, and Rb7) (Table 3). Magnesium (Mg) concentration in Rb7 was obtained to be the highest concentration (29.90 mg/100 g) than the rest ones. Zinc (Zn) concentrations were varied from 0.53–0.65 mg/100 g in all formulated raw complementary flour blends.

### 3.4. Mineral Composition of Extruded and Drum-Dried Complementary Flour Blends

Mineral compositions of extruded and drum-dried complementary flour blends are explained in Tables 4 and 5, respectively.

As depicted in Tables 4 and 5, the Ca concentrations varied from 3.88–4.48 mg/100 g and 13.27–22.55 mg/100 g in all extruded and drum-dried complementary flour blends, respectively. The recommended daily intake of Ca for infants ranges from 210–500 mg/day [20, 68]. In this study, the obtained Ca contents in all extruded and drum-dried complementary flour blends were below the recommended range for infants. The Ca concentration of the commercial famix is 80 mg/100 g, which is higher than the extruded and drum-dried complementary foods. This may be due to the addition of premix minerals in famix complementary food [60]. The values of Ca in all drum-dried complementary flour blends are higher than that of reported value (13.1 mg/100 g) for maize-soybean blend [69] but extruded products are not, and both extruded and drum-dried products are lower than the reported values (98.2 mg/100 g) for maize, sorghum, millet, and soybean flour blend [70]. The Ca content in the current study that obtained in DDb1 (22.55 mg/100 g) is similar to the values (22 mg/100 g) of Nutrend-commercial complementary food from Nigeria [71]. The Ca content of DDb1 (22.55 mg/100 g) is similar with the reported value (23.91 mg/100 g) for OFSP, millet, and soybean flour blend of complementary food [20].

The recommended daily intake of Fe for infants aged 6–8 months is 0.27 mg, 9–11 months is 11 mg, and 12–23 months is 7 mg [23]. Fe contents in all extruded and drum-dried complementary flour blends meet the recommended daily intake of infants aged at 6–8 months but did not meet the need of the rest two age groups of infants. The Fe concentration of the commercial famix is 6 mg/100 g, which is higher than the current complementary foods [60]. Fe contents of DDb5 (1.00 mg/100 g) are similar to the values (1.00 mg/100 g) of Nutrend-commercial complementary food in Nigeria [71]. Fe content of DDb1 (1.68 mg/100 g) is found to be similar with the reported values (1.95 mg/100 g) of OFSP, millet, and soybean flour blend of complementary food [20].

The recommended K concentration in infant foods is 60–160 mg/100 g [68]. Therefore, the K concentrations in all extruded and drum-dried complementary flour blends (varied from 74.75–88.43 mg/100 g) are found to meet the required range for infant foods (Tables 4 and 5). The K contents of all extruded and drum-dried products are found to be lower than the reported value (234.81 mg/100 g) for OFSP, millet, and soybean flour blend of complementary food [20].

The recommended intake of Mg for infants ranges from 30–80 mg/day [68]. The Mg contents of all extruded and drum-dried products are found to be lower than the recommended amount and reported value (56.87 mg/100 g) for OFSP, millet, and soybean flour blend of complementary food [20].

The recommended daily intake of Zn for infants aged at 6–8 months, 9–11 months, and 12–23 months is 2, 3, and 3 mg/day, respectively [23]. In this study, the obtained Zn concentrations in all formulated complementary flour blends are found to be below the recommended daily intake of infants aged between 6 and 23 months. The need of infants aged 6–8 months can be fulfilled at 3 servings per day (Tables 4 and 5). Zn contents of all extruded and drum-dried complementary flour blends are approximately similar with reported values (0.89 mg/100 g) for OFSP, millet, and soybean flour blend of complementary food [20].

### 3.5. Antinutritional Compositions of Formulated Complementary Flour Blends

Antinutritional compositions of formulated complementary flour blends are depicted in Table 6.

As shown in Table 6, the amount of phytate in all raw complementary flour blends varied from 72.99–124.78 mg/100 g. There was no significant difference (*P* > 0.05) between phytate contents in all raw blends except Rb2. Rb2 had the greatest amount of phytate (124.78 mg/100 g), and Rb7 had the least phytate content (72.99 mg/100 g) corresponding to others. In terms of condensed tannin content, Rb5 and Rb2 had the largest value of 715 mg/100 g and 702 mg/100 g, respectively. The tannin content in Rb1 was found to be the smallest value (499 mg/100 g) than others. The amount of oxalate in all raw complementary flour blends varied from 210.10–399.19 mg/100 g.

The foods with low phytate content are recommended for infants and all consumers. Therefore, the phytate content of the current products is very low when compared with the recommended daily intake of phytate from complementary foods (300–500 mg/day) [72–74]. Phytate levels (varied from 64.64–102.57 mg/100 g) in extruded and drum-dried products are found to be lower than the reported composite flour blends of wheat, anchote, and soybeans (varied from 145.59–245.37 mg/100 g) [55].

Condensed tannin contents of all extruded and drum-dried products except Eb2 (567 mg/100 g) and DDb2 (589 mg/100 g) could be considered safe because the tannin contents were found to be lower than the recommended daily intake for man (560 mg). Condensed tannin contents (varied from 209–589 mg/100 g) were found to be higher than the reported composite flour blends of wheat, anchote, and soybeans (varied from 101.89–194.30 mg/100 g) [55].

Diets low in calcium and high in oxalates are not recommended but the unusual/rare consumption of high oxalate foods, as part of a nutritious diet does not cause any particular problem [75]. Consuming of 2–5 g of oxalate per day, this is assumed to be the toxic level for humans [76]. Hence, oxalate contents in all the extruded and drum-dried products were found in a safe level. However, there is no defined and specific guideline on recommended daily intake of phytate, tannin, and oxalate as protein, fat, energy, and some elements for infants aged 6–23 months [77]. Codex Alimentarius Commissions should provide specific guidelines on acceptable daily intake of antinutrients for infants and young children.

### 3.6. Viscosities of Drum-Dried Complementary Flour Blends and Control Sample Gruels

Viscosities of drum-dried complementary flour blends and control sample gruels are depicted in Table 7.

As explained in Table 7, a significantly (*P* < 0.05) higher viscosity value (2,400 cP) was observed in the control sample (*faffa*). Within the seven formulated gruels, the values of viscosities were observed to be increased as the proportion of OFSP increased as shown in Table 7. Typically high viscosity (and thus, low energy density) of cereal gruels consumed by infants in many developing countries has been viewed as a cause of low energy intake because of more dilutions to reduce viscosity of gruels [66]. A viscosity value of 1,000–3,000 cP is suggested to be appropriated consistency of complementary foods that is easy to swallow for infants and young children [78, 79].

In the current study, the values of viscosities for the seven formulated gruels were found to be in the optimum range (Table 7). The observed increment in the viscosity of formulated gruels when the proportion of OFSP flour increased in the current study may be due to the result of starch degradation, which caused by the action of high heat and moisture content that applied during the drum-drying process. This high heat degrades starch into dextrin and maltose rather than melting (gelatinizing) it, which increases the solubility of OFSP flour particles [80]. This leads to a decrease in particle size of OFSP flour in cooked gruels. Then, the numbers of particles increase when particle size decreases. As a result of this, the number of interactions between particles increases and then causes to an overall increase in viscosity of gruels as the OFSP proportion increased.

### 3.7. Sensory Evaluation of Gruels

The results of sensory evaluation of gruels are depicted in Table 8.

As shown in Table 8, the preference of the drum-dried complementary food formulated, with reference to all parameters given, was varied from “like slightly (5)” to “Like moderately (6)”. Preference for color of samples was the highest in blend-5 and blend-2, followed by *faffa* (commercial complementary food). The three blends, blend-6, blend-4, and blend-3, were relatively the least preferred. The preference for the smell of samples was the highest in *faffa* and blend-5, followed by blend-1. The preference for sweetness of the samples was highest in blend-1, *faffa*, blend-2, and blend-5. The mouthfeel/texture of complementary foods (blend-1, 2, 3, 7, and 5) was liked moderately as a control sample (*faffa*) and blend-4 and 6 were liked slightly. Preference for aftertaste of samples was highest in *faffa*, blend-7, blend-3, blend-6, and blend-5. A bitter aftertaste was relatively observed by panelists in blend-1 and blend-2. This may be due to the formation of a bitter compound of OFSP known as ipomeamarone, which may be resulted by dry heat application during the end of the process [81]. This compound may have been more distinct in flour blends with relatively high OFSP content and may have resulted in the relatively least preference for blend-1 and blend-2 gruels. According to overall acceptability, blend-5, blend-1, and blend-3 were the most preferred ones relative to the overall acceptability of *faffa*. The others (blend-2 and blend-7) were liked moderately, and blend-4 and blend-6 were liked slightly.

The complementary foods that developed from flour blend of OFSP, BT, and DRKB were comparable with control commercial complementary food (*faffa*) in terms of almost all sensory properties according to panelists' assessment. The researchers have been reported that the drum-drying process is suitable for the development of complementary foods and baby cereals [82]. Therefore, the drum dryer could be used to develop food products with good sensorial attributes [83].

## 4. Conclusions

In this study, the complementary food products were formulated from flour blends of OFSP, BT, and DRKB to complement the nutritional need of infants aged 6–23 months. From the formulated food products, drum-dried products contained significantly higher macronutrients than extruded products. The extrusion process was more effective in phytate and tannin content reduction than the drum-drying process. The drum-drying process was more effective in oxalate content reduction than the extrusion process. Drum-dried products DDb1, DDb4, and DDb5 were contained 5.97%, 12.89%, and 14.08% of protein, respectively. Their available carbohydrate and energy content were varied from 69.30–80.45% and 347.20–356.57 Kcal/100 g, respectively. Their antinutritional content was below the toxic level. Moreover, sensory properties of gruels of DDb1 and DDb5 were moderately liked as *faffa* and DDb4 was slightly liked. These newly formulated food products can meet the macronutritional (except fat) needs of infants. However, they do not meet the recommended intake of minerals (except potassium) for infants. Hence, optimization with appropriate micronutrient dense foodstuffs will be crucial. The proper optimization of these complementary food products can provide nutritious foods that are suitable for complementing breast milk in all aspects. Therefore, consumption of these complementary foods can be used to combat the problem of protein-energy malnutrition among infants in Ethiopia and other developing countries.

## Figures and Tables

**Table 1:** Proximate composition of OFSP, BT, and DRKB per 100 g of flour.

Samples	Moisture content (%)	Ash (%)	Crude protein (%)	Crude fat (%)	Crude fiber (%)	Carbohydrate (%)	Energy (Kcal/100 g)
OFSP	7.32 ± 0.04^a^	2.91 ± 0.04^b^	2.25 ± 0.09^c^	0.62 ± 0.01^d^	3.69 ± 0.03^e^	83.21 ± 0.04^a^	347.42 ± 0.05^a^
BT	11.07 ± 0.76^b^	2.32 ± 0.04^c^	9.49 ± 0.13^d^	2.77 ± 0.01^e^	3.48 ± 0.02^f^	70.87 ± 0.19^b^	346.37 ± 0.11^b^
DRKB	11.33 ± 0.16^b^	3.93 ± 0.02^d^	18.01 ± 0.22^e^	1.76 ± 0.03^f^	4.47 ± 0.02^g^	60.50 ± 0.09^c^	329.88 ± 0.11^c^

Means with the same superscript letters within the same column are not significantly different (*P* > 0.05). All values are means ± SD of the triplicate (*n* = 3).

**Table 2:** Proximate compositions of raw, extruded, and drum-dried complementary flour blends.

Sample	Moisture (%)	Ash (%)	Crude protein (%)	Crude fat (%)	Crude fiber (%)	Available carbohydrate (%)	Energy (Kcal/100 g)
Rb1	9.88 ± 0.01^j^	3.04 ± 0.02^abc^	6.48 ± 0.09^f^	1.63 ± 0.28^cde^	3.21 ± 0.05^m^	75.76 ± 0.09^k^	343.63 ± 0.15^g^
Eb1	4.92 ± 0.09^a^	4.02 ± 0.06^cd^	4.82 ± 0.18^cd^	0.18 ± 0.00^a^	2.10 ± 0.03^cde^	83.96 ± 0.07^t^	356.74 ± 0.08^s^
DDb1	6.20 ± 0.03^c^	3.47 ± 0.01^abcd^	5.97 ± 0.00^e^	1.21 ± 0.03^b^	2.70 ± 0.02^ij^	80.45 ± 0.02^p^	356.57 ± 0.01^r^
Rb2	10.22 ± 0.02^k^	3.30 ± 0.01^abcd^	6.29 ± 0.09^f^	1.99 ± 0.41^f^	3.18 ± 0.08^lm^	75.02 ± 0.12^j^	343.15 ± 0.12^f^
Eb2	5.88 ± 0.10^b^	3.78 ± 0.02^e^	4.79 ± 0.05^cd^	0.17 ± 0.01^a^	2.02 ± 0.01^cd^	83.36 ± 0.04^s^	354.13 ± 0.03^q^
DDb2	7.68 ± 0.03^de^	3.55 ± 0.04^abcd^	5.88 ± 0.03^e^	1.67 ± 0.04^cde^	2.67 ± 0.02^i^	78.55 ± 0.03^n^	352.75 ± 0.03^p^
Rb3	10.46 ± 0.04^l^	2.98 ± 0.04^ab^	4.90 ± 0.16^d^	1.89 ± 0.30^ef^	3.48 ± 0.04^n^	76.29 ± 0.12^l^	341.77 ± 0.19^d^
Eb3	7.58 ± 0.11^d^	3.82 ± 0.03^abcd^	3.65 ± 0.10^a^	0.24 ± 0.01^a^	2.28 ± 0.03^efg^	82.43 ± 0.05^r^	346.48 ± 0.05^k^
DDb3	7.82 ± 0.01^e^	3.39 ± 0.02^abcd^	4.45 ± 0.02^b^	1.59 ± 0.03^cd^	2.98 ± 0.01^kl^	79.77 ± 0.02^o^	351.19 ± 0.02^n^
Rb4	10.63 ± 0.16^m^	3.22 ± 0.03^abcd^	13.13 ± 0.49^jk^	1.67 ± 0.23^cde^	3.47 ± 0.07^n^	67.88 ± 0.20^c^	339.07 ± 0.31^a^
Eb4	7.52 ± 0.05^d^	3.80 ± 0.06^abcd^	11.44 ± 0.10^g^	0.28 ± 0.01^a^	2.42 ± 0.02^fgh^	74.54 ± 0.05^i^	346.44 ± 0.05^ij^
DDb4	8.02 ± 0.02^f^	3.53 ± 0.02^abcd^	12.89 ± 0.01^j^	1.55 ± 0.05^c^	2.90 ± 0.03^jk^	71.11 ± 0.03^f^	349.95 ± 0.03^m^
Rb5	10.71 ± 0.09^m^	3.29 ± 0.04^abcd^	14.25 ± 0.16^m^	1.64 ± 0.04^cde^	3.01 ± 0.08^mn^	67.10 ± 0.08^a^	339.76 ± 0.09^b^
Eb5	8.70 ± 0.04^h^	3.95 ± 0.04^bcd^	12.41 ± 0.10^i^	0.31 ± 0.01^a^	1.97 ± 0.02^bc^	72.66 ± 0.04^g^	343.07 ± 0.05^f^
DDb5	8.97 ± 0.01^i^	3.65 ± 0.03^abcd^	14.08 ± 0.03^m^	1.52 ± 0.01^c^	2.48 ± 0.03^ghi^	69.30 ± 0.02^d^	347.20 ± 0.02^l^
Rb6	11.56 ± 0.14^n^	3.12 ± 0.02^abcd^	13.77 ± 0.19^l^	1.74 ± 0.01^cde^	2.49 ± 0.20^ghi^	67.32 ± 0.11^b^	340.02 ± 0.10^c^
Eb6	8.02 ± 0.08^f^	4.05 ± 0.05^d^	11.82 ± 0.09^h^	0.16 ± 0.01^a^	1.67 ± 0.03^a^	74.28 ± 0.05^h^	345.84 ± 0.05^h^
DDb6	8.58 ± 0.02^h^	3.62 ± 0.05^abcd^	13.21 ± 0.02^k^	1.62 ± 0.02^cd^	2.06 ± 0.05^cde^	70.91 ± 0.03^e^	351.06 ± 0.02^n^
Rb7	11.45 ± 0.25^n^	2.83 ± 0.02^a^	6.42 ± 0.13^f^	1.82 ± 0.02^def^	2.50 ± 0.02^hi^	74.98 ± 0.09^j^	341.98 ± 0.08^e^
Eb7	8.03 ± 0.15^f^	3.94 ± 0.04^bcd^	4.61 ± 0.14^bc^	0.26 ± 0.01^a^	1.78 ± 0.03^ab^	81.38 ± 0.07^q^	346.30 ± 0.07^i^
DDb7	8.34 ± 0.03^g^	3.45 ± 0.03^abcd^	5.94 ± 0.01^e^	1.70 ± 0.05^cde^	2.23 ± 0.01^def^	78.34 ± 0.03^m^	352.42 ± 0.03^o^

Abbreviations: R: raw; E: extruded; DD: drum dried; and b: blend. Means with the same superscript letters within the same column are not significantly different (*P* > 0.05). All values are mean ± SD of the triplicate (*n* = 3).

**Table 3:** Mineral composition (mg/100 g) of raw complementary flour blends.

Sample	Ca (mg/100 g)	Fe (mg/100 g)	K (mg/100 g)	Mg (mg/100 g)	Zn (mg/100 g)
Rb1	30.56	2.00	86.52	10.76	0.59
Rb2	24.77	0.95	89.66	12.24	0.53
Rb3	24.77	1.02	87.87	10.84	0.58
Rb4	20.13	0.95	83.26	11.45	0.58
Rb5	17.26	1.03	88.85	11.90	0.63
Rb6	16.14	1.25	80.48	12.68	0.62
Rb7	18.37	1.32	77.50	29.90	0.65

**Table 4:** Mineral composition of extruded complementary flour blends.

Sample	Ca (mg/100 g)	Fe (mg/100 g)	K (mg/100 g)	Mg (mg/100 g)	Zn (mg/100 g)
Eb1	4.07 (86.67)	0.85 (57.66)	85.74 (0.90)	9.95 (7.46)	0.57 (3.88)
Eb2	3.88 (84.31)	0.42 (56.23)	85.63 (4.50)	10.65 (12.94)	0.52 (1.58)
Eb3	4.22 (82.98)	0.41 (59.84)	86.38 (1.70)	9.10 (16.06)	0.49 (15.94)
Eb4	4.48 (77.74)	0.47 (50.23)	78.87 (5.28)	8.68 (24.22)	0.50 (13.60)
Eb5	4.06 (74.47)	0.51 (49.88)	86.64 (2.49)	10.88 (8.57)	0.59 (7.54)
Eb6	4.19 (74.04)	0.51 (59.32)	75.79 (5.88)	12.10 (4.53)	0.59 (4.65)
Eb7	4.29 (76.66)	0.55 (57.95)	74.75 (3.55)	27.77 (7.15)	0.57 (12.30)

Numbers aside indicates the percentage of reduction over the value of the corresponding formulated raw complementary flour blends.

**Table 5:** Mineral composition of drum-dried complementary flour blends.

Sample	Ca (mg/100 g)	Fe (mg/100 g)	K (mg/100 g)	Mg (mg/100 g)	Zn (mg/100 g)
DDb1	22.55 (26.20)	1.68 (15.82)	86.32 (0.23)	10.76 (0.02)	0.57 (3.55)
DDb2	16.77 (32.28)	0.75 (20.94)	88.43 (1.37)	12.01 (1.85)	0.52 (0.76)
DDb3	17.10 (30.94)	0.98 (3.76)	87.47 (0.45)	10.79 (0.42)	0.55 (5.07)
DDb4	15.44 (23.26)	0.62 (34.89)	82.53 (0.88)	10.99 (4.06)	0.58 (0.36)
DDb5	13.75 (20.33)	1.00 (2.29)	87.05 (2.03)	11.86 (0.28)	0.61 (4.34)
DDb6	13.27 (17.81)	1.09 (13.14)	77.76 (3.37)	12.44 (1.86)	0.60 (3.44)
DDb7	14.78 (19.55)	1.03 (21.67)	77.12 (0.49)	29.86 (0.16)	0.61 (4.97)

Numbers aside indicates the percentage reduction over the value of the corresponding formulated raw complementary flour blends.

**Table 6:** Antinutritional compositions of raw, extruded, and drum-dried complementary flour blends.

Sample	Phytate (mg/100 g)	Tannin (mg/100 g)	Oxalate (mg/100 g)
Rb1	94.64 ± 1.94^de^	499 ± 0.06^m^	252.12 ± 0.00^h^
Eb1	89.36 ± 1.20^cde^	384 ± 0.08^h^	189.09 ± 0.00^d^
DDb1	89.89 ± 2.12^cde^	406 ± 0.02^i^	185.94 ± 0.01^c^
Rb2	124.78 ± 2.96^f^	702 ± 0.16^t^	210.1 ± 0.36^f^
Eb2	101.39 ± 1.22^e^	567 ± 0.18^p^	168.08 ± 0.36^b^
DDb2	102.57 ± 1.03^e^	589 ± 0.01^q^	150.00 ± 0.02^a^
Rb3	77.55 ± 2.67^abcd^	685 ± 0.23^s^	294.14 ± 0.36^l^
Eb3	76.84 ± 0.68^abc^	349 ± 0.23^f^	273.13 ± 0.36^j^
DDb3	77.09 ± 0.09^abc^	417 ± 0.01^j^	268.34 ± 0.00^i^
Rb4	82.36 ± 1.01^bcd^	624 ± 0.10^r^	252.12 ± 0.63^h^
Eb4	67.19 ± 1.62^ab^	233 ± 0.08^b^	210.10 ± 0.36^f^
DDb4	67.53 ± 1.45^ab^	306 ± 0.02^d^	209.49 ± 0.01^e^
Rb5	82.73 ± 2.73^bcd^	715 ± 0.23^u^	375.17 ± 0.36^o^
Eb5	73.08 ± 0.25^abc^	436 ± 0.18^k^	294.14 ± 0.36^l^
DDb5	73.18 ± 1.19^abc^	439 ± 0.03^l^	279.61 ± 0.01^k^
Rb6	79.22 ± 0.52^abcd^	557 ± 0.45^o^	273.13 ± 0.36^j^
Eb6	66.36 ± 1.47^ab^	347 ± 0.43^e^	252.12 ± 0.00^h^
DDb6	66.48 ± 0.33^ab^	376 ± 0.04^g^	245.11 ± 0.00^g^
Rb7	72.99 ± 0.10^abc^	555 ± 0.44^n^	399.19 ± 0.36^p^
Eb7	64.64 ± 2.24^ab^	209 ± 0.25^a^	336.16 ± 0.36^n^
DDb7	64.66 ± 1.33^a^	259 ± 0.06^c^	315.92 ± 0.01^m^

Means with the same superscript letters within the same column are not significantly different (**P** > 0.05). All Values are mean ± SD of the triplicate (**n** = 3).

**Table 7:** Viscosities of drum-dried complementary flour blends and control sample gruels.

Code of samples	Viscosity (cP)
505 (blend-5)	1, 397 ± 1.73^b^
202 *(faffa)*	2, 400 ± 2.00^h^
606 (blend-6)	1, 289 ± 1.22^a^
404 (blend-4)	2, 081 ± 3.00^c^
303 (blend-3)	2, 207 ± 0.22^d^
101 (blend-1)	2, 274 ± 0.12^g^
707 (blend-7)	2, 225 ± 0.02^e^
002 (blend-2)	2, 258 ± 0.03^f^

Means with the same superscript letters within the same column are not significantly different (*P* > 0.05). All values are mean ± SD of the triplicate (*n* = 3).

**Table 8:** Sensory evaluation of gruels prepared from drum-dried complementary flour blends and control sample.

Sample	Color	Smell	Taste/sweetness	Mouth feel	After taste	Overall acceptability
505	6.25	6.06	6.00	5.75	5.75	6.13
202	6.19	6.50	6.13	5.94	5.81	6.19
606	5.25	5.69	5.56	4.88	5.50	5.31
404	5.13	5.06	5.69	5.00	5.19	5.00
303	5.21	5.98	5.71	5.89	5.77	6.09
101	5.63	6.01	6.23	6.15	5.03	6.11
707	5.57	5.66	5.60	5.88	5.79	5.93
002	6.21	5.87	6.10	5.96	5.10	5.78

Scale: 1-dislike very much, 2-dislike moderately, 3-dislike slightly, 4-neither like nor dislike, 5-like slightly, 6-like moderately, and 7-like very much.

## Data Availability

The data used to support the findings of this study are included within the article.
